# Effectiveness of music therapy as an aid to neurorestoration of children with severe neurological disorders

**DOI:** 10.3389/fnins.2015.00427

**Published:** 2015-11-04

**Authors:** Maria L. Bringas, Marilyn Zaldivar, Pedro A. Rojas, Karelia Martinez-Montes, Dora M. Chongo, Maria A. Ortega, Reynaldo Galvizu, Alba E. Perez, Lilia M. Morales, Carlos Maragoto, Hector Vera, Lidice Galan, Mireille Besson, Pedro A. Valdes-Sosa

**Affiliations:** ^1^Laboratory of Neuroinformation, School of Life Sciences, University of Electronic Sciences and Technology of ChinaChengdu, China; ^2^Centro Internacional de Restauracion NeurologicaHabana, Cuba; ^3^Centro de Neurociencias de CubaHabana, Cuba; ^4^Laboratoire de Neurosciences Cognitives, Centre National de la Recherche Scientifique and Marseille UniversitéMarseille, France

**Keywords:** rehabilitation, children, ERPs, music therapy, neurological disorders

## Abstract

This study was a two-armed parallel group design aimed at testing real world effectiveness of a music therapy (MT) intervention for children with severe neurological disorders. The control group received only the standard neurorestoration program and the experimental group received an additional MT “Auditory Attention plus Communication protocol” just before the usual occupational and speech therapy. Multivariate Item Response Theory (MIRT) identified a neuropsychological status-latent variable manifested in all children and which exhibited highly significant changes only in the experimental group. Changes in brain plasticity also occurred in the experimental group, as evidenced using a Mismatch Event Related paradigm which revealed significant post intervention positive responses in the latency range between 308 and 400 ms in frontal regions. LORETA EEG source analysis identified prefrontal and midcingulate regions as differentially activated by the MT in the experimental group. Taken together, our results showing improved attention and communication as well as changes in brain plasticity in children with severe neurological impairments, confirm the importance of MT for the rehabilitation of patients across a wide range of dysfunctions.

## Introduction

The rehabilitation of children with severe neurological disorders is an area of great current interest (Katona, 1989). Different therapies have been designed to enhance neural plasticity and thus promote recovery of function (Gordon and Di Maggio, 2012). A recent review of rehabilitation of children with acquired brain injury (Forsyth and Basu, 2015) argues that improved results might follow from “greater doses” of treatment that might produce more extensive compensatory brain plasticity. However, an alternative to simply increasing the amount of a specific intervention might be to enhance standard treatments by using different adjunct procedures. One such potential adjunct procedure might be Music Therapy (MT) (Bruscia, 1998). The basic idea is to use music interventions to improve non-musical abilities (e.g., social, academic, communication) that are deficient in individual patients (see Brown and Jellison, 2012). A recent comprehensive review of current studies, and of the impact of MT in neuropediatric settings, recommends MT for general use in a wide variety of disorders (see Yinger and Gooding, 2014).

Rather than simple exposure to music or “music listening” or even “music training” (playing a musical instrument), a more precise definition of the concept of MT is the use of music to modify brain processes by engaging the attention and interest of the subject and by confirmation of this engagement effect and its consequences. This concept of MT in neurological settings has been documented, standardized and given a neuroscientific basis by Thaut and Mcintosh (2010) and Thaut and Hoemberg (2014)—creating a field known as neurologic music therapy (NMT). The Rational Scientific Mediating Model (Thaut, 2005) provides a systematic epistemology for translational research in music and rehabilitation. Importantly, NMT protocols may be applied not only to adults but also to children with a wide spectrum of pathologies and neuropsychological impairments. The therapy described later in the article may be considered as a variant of NMT.

While there is considerable support for the *efficacy* of MT in specific childhood disorders, there are also a number of shortcomings in the field which question its more general application (see Mrázová and Celec, 2010). For example, the Cochrane Collection reviews of Randomized Clinical Trials (RCT) have shown a beneficial effect of MT on Autistic Spectrum Disorder (ASD) compared with a placebo treatment (Gold et al., 2006; Geretsegger et al., 2014). On the other hand, to the best of our knowledge there are few studies that have evaluated the “real world” *effectiveness* of MT for a wider range of neuropediatic disorders using appropriate behavioral and physiological outcome measures. The main objective of the current study is therefore to investigate whether MT can indeed be more widely applied to such disorders. In view of the widespread effect of music (listening or production) on different brain structures involved in cognitive, sensorimotor and emotional processing (Koelsch, 2009), we hypothesized that MT might produce greater beneficial effects than standard neurorestoration therapy alone.

An important issue in MT is to provide objective measures of changes in brain plasticity. In spite of growing neuroimaging evidence for the effects of music training on brain plasticity (Kraus and Chandrasekaran, 2010), music-evoked emotions (Koelsch, 2014), and reward value (Zatorre, 2013), there is a paucity of studies using these techniques in MT trials (Stegemöller, 2014). Indeed, most MT trials only record behavioral outcomes—an issue highlighted in a recent review that concludes that pediatric neuroimaging will play a major role in the future but requires further intensive study (Yinger and Gooding, 2014). Unfortunately, this objective is limited by the expensive, intrusive (and certainly not widely applicable) character of most neuroimaging methods.

A viable electrophysiological alternative for measuring brain plasticity is the family of Mismatch Responses (MMR)—the differential change of event related brain potentials (ERP) to “deviant stimuli” embedded in a sequence of “standard stimuli.” The MMR are very sensitive biomarkers both for normal processes and brain disorders (Näätänen et al., 2007, 2011; Lepistö et al., 2008; Kujala and Näätänen, 2010). The best known of the MMR is the “*early Mismatch Negativity”* (MMN) (Näätänen et al., 1978). However, the MMR family also includes a “*late discriminatory negativity*” (LDN) (Korpilahti et al., 2001) and a “*positive mismatch response*” (pMMR) (Dehaene-Lambertz and Dehaene, 1994). These ERP responses have been used in children to gauge brain maturation (Liu et al., 2014) and to study specific childhood neurological disorders. They are altered in specific language impairment (Bishop, 2007; Hommet et al., 2009), reflect risk of familial dyslexia (Maurer et al., 2003), and are even predictive of reading ability (Maurer et al., 2009). The MMN have been shown to change with musical training (François et al., 2013; Chobert et al., 2014; Putkinen, 2014) although, to the best of our knowledge, they have not been used to date to evaluate changes in brain plasticity during MT in neurorestorative settings.

Thus, the overall objective of the current research is to address two specific questions:

- Is MT, in addition to standard neurorestoration therapy, effective in producing further improvements in the cognitive performance of children with diverse and severe neurological disorders?
- Do ERP Mismatch Responses identify MT-specific changes in brain plasticity after therapy?

## Materials and methods

### Study design

**Figure 1:**
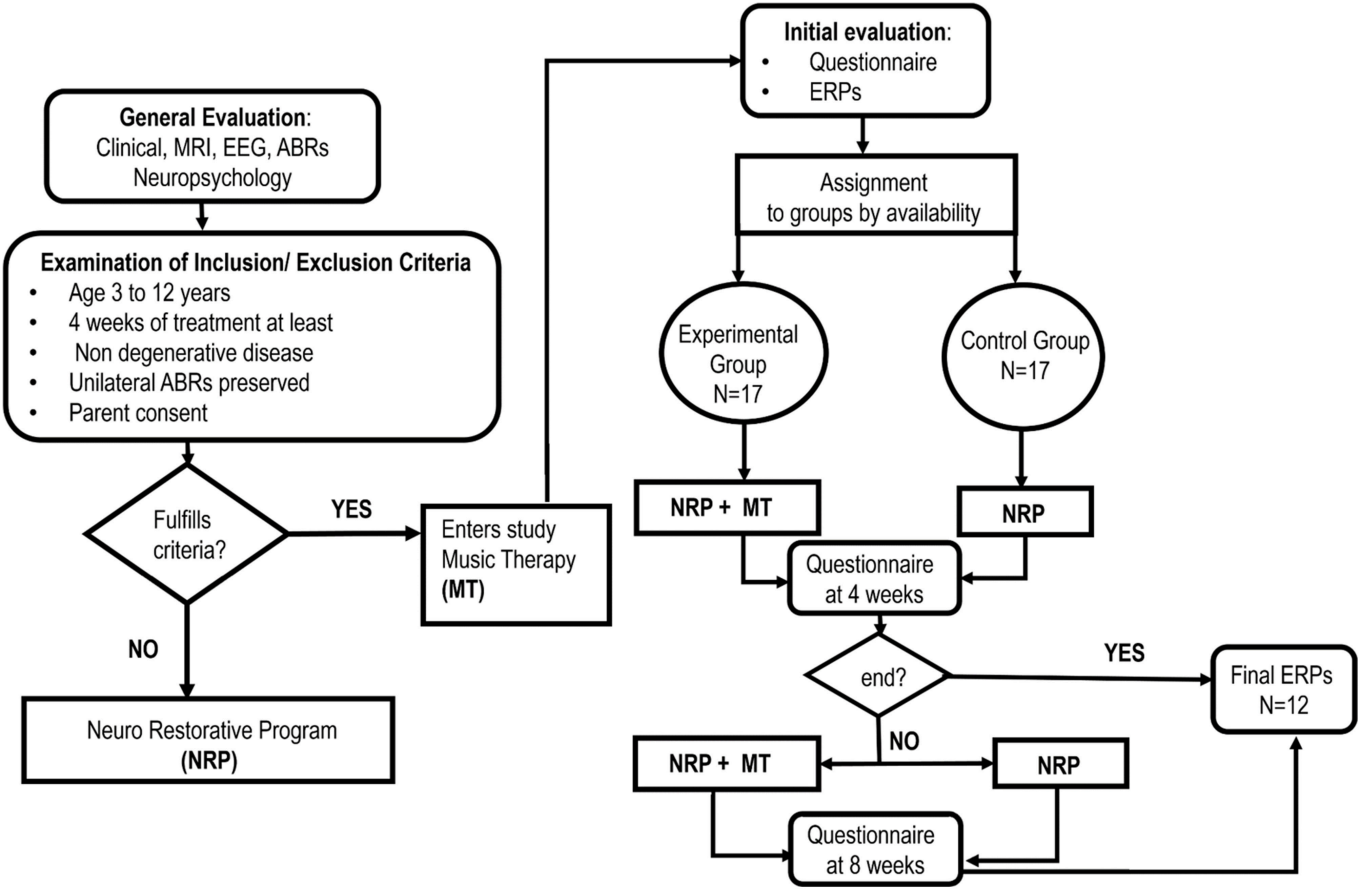
**Study flow chart**.

The study was a two-arm parallel group design in which a MT group (experimental) was compared to a control group. The overall design is shown in Figure 1. Both groups received a standard neurorestoration program (NRP) but the experimental group, as described below, received additional music therapy.

Convenience sampling was carried out from a population of 252 patients admitted consecutively to the Neuropediatric Clinic at CIREN (www.ciren.cu) between January 2013 and July 2014 for neuro-rehabilitation treatment. It should be noted that all the children who were referred to the intensive rehabilitation program had significant problems in motor, cognitive, and, in particular communication, abilities (See **Table 2**)

The inclusion criteria were: participation in the standard NRP for at least 4 weeks; ages ranging from 3 to 12 years; having a preserved unilateral auditory response (recorded using Auditory Brainstem Responses) and written parental consent. The only exclusion criteria were the presence of a neurodegenerative disease. This resulted in a sample of 34 children, 25 that suffered from Static Lesions of the Central Nervous System of prenatal and/or perinatal origins expressed in the context of cerebral palsy and/or cognitive disorders and 9 other neurological disorders (two children with spinal cord lesions). Table 1 provides a full clinical and demographic details of the patients.

**Table 1:** **Clinical characteristics of the samples**.

**Subject**	**Sex**	**Age years/month**	**Duration of therapy (Weeks)**	**Diagnosis**	**Expressed as**	**Magnetic resonance imaging**	**Electro-encephalogram**	**Auditory brainstem response**
**A. EXPERIMENTAL GROUP**								
1	F	6.85	8	Static lesion nervous system	Cognitive deficit	Dysplasia in left temporal region.	Epileptiform activity	Normal
2	F	9.93	8	Static lesion nervous system	Dyskinetic cerebral palsy	Cortical atrophies in insular region. Vascular lesion at left occipito- mesial area. Caudate nucleus lesion.	Not indicated	Unilateral
3	F	9.22	4	Static lesion nervous system	Cognitive deficits	Bilateral atrophy of hippocampus.	Not indicated	Normal
4	M	8.79	8	Static lesion nervous system	Spastic cerebral palsy	Cortical heterotopias deforming lateral ventricles at oval centers. Hypoxia signs.	Not indicated	Unilateral
5	M	5.43	8	Static lesion nervous system	Spastic cerebral palsy	Left fronto-parietal sulci absents.	Epileptiform activity	Unilateral
6	M	7.67	8	Static lesion nervous system	Spastic cerebral palsy	Subcortical lesions adjacent to upper lateral ventricles. Mild bilateral hippocampal atrophy. Pineal gland Cyst.	Not indicated	Normal
7	F	11.6	4	Static lesion nervous system	Cerebral palsy	Cortical heterotopias deforming lateral ventricles. Right frontal atrophy.	Not indicated	Normal
8	M	8	4	Neurodevelopmental disorder	Non-verbal learning disability	Mild right fronto-temporal atrophy.	Epileptiform activity	Normal
9	M	4	4	Static lesion nervous system	Language impairment	Normal.	Normal	Normal
10	M	3.2	8	Static lesion nervous system	Cerebral palsy	Hypoxic lesions in periventricular area.	Not indicated	Normal
11	M	3.1	8	Static lesion nervous system	Cerebral palsy	Subcortical lesions adjacent to lateral ventricles. Mild atrophy left temporal lobe. Hypoplasia Corpus Callosum.	Not indicated	Normal
12	F	7	4	Static lesion nervous system	Cognitive deficit	Normal.	Slow waves	Normal
13	F	6	8	Spinal cord lesion	Paraplegia	Normal.	Not indicated	Normal
14	F	11	8	Idiopathic dystonia	Motor symptoms-	Cortical atrophy (temporal lobe). *Cavum vergae*. Brainstem hypoplasia.	Not indicated	Normal
15	M	7	8	Neurofibromatosis	Motor symptoms-	Tumor. Frontal atrophy.	Epileptiform activity	Unilateral
16	M	4.2	4	Static lesion nervous system	Cognitive and language impairments	Bilateral atrophy hippocampus.	Epileptiform activity	Normal
17	M	5.8	4	Autistic spectrum disorder	Mioclonic epilepsy and attention deficits disorders hyperactivity	Left occipital lesion related to heterotopia area.	Epileptiform activity	Unilateral
**B. CONTROL GROUP**								
1	F	9.48	8	Static lesion nervous system	Dyskinesia cerebral palsy	Cortical atrophy. *Cavum vergae*.	Normal	Normal
2	M	12	8	Spinal cord lesion	Paraplegia	Normal.	Not indicated	Normal
3	F	4.71	8	Static lesion nervous system	Cognitive deficits	Left occipital lesion (heterotopia).	Epileptiform activity	Normal
4	F	3.99	4	Autistic disorder spectrum	Rhett syndrome	Mild cortical atrophy more evident at temporal lobes.	Epileptiform activity	Unilateral
5	M	9.64	4	Static lesion nervous system	Spastic cerebral palsy	Normal.	Slow waves	Normal
6	M	3	8	Static lesion nervous system	Spastic cerebral palsy	Periventricular heterotopias. Corpus callosum hipoplasia.	Not indicated	Normal
7	M	4.96	4	Static lesion nervous system	Language disorder	Diminished cranial diameter. Temporal operculum atrophy.	Epileptiform activity	Normal
8	M	12	4	Static lesion nervous system sequel to hypoxia	Cognitive deficit	Generalized cortical atrophy. Hypoxic lesion at basal ganglia.	Epileptiform activity	Unilateral
9	M	4.6	4	Static lesion nervous system	Spastic cerebral palsy	Periventricular heterotopias.	Epileptiform activity	Normal
10	M	12	8	Static lesion nervous system sequel to hypoxia	Cerebral palsy	Periventricular heterotopias, right hippocampal atrophy.	Not indicated	Normal
11	F	12	4	Static lesion nervous system sequel to hypoxia	Cerebral palsy	Periventricular heterotopias.	Not indicated	Normal
12	F	10	4	Static lesion nervous system	Spastic cerebral palsy	Cortical heterotopias.	Not indicated	Normal
13	F	12.4	4	Friederich's ataxia	Motor symptoms	Cerebellar atrophy.	Not indicated	Normal
14	F	12.2	4	Static lesion nervous system	Spastic cerebral palsy	Cortical atrophy.	Not indicated	Normal
15	M	4	4	Mielomeningocele	Spastic paraparesis	Mielomeningocele malformation.	Not indicated	Normal
16	M	3.10	4	Static lesion nervous system	Spastic cerebral palsy	Periventricular heterotopias and hypoxic lesions.	Not indicated	Normal
17	M	4.7	4	Static lesion nervous system	Cerebral palsy	Periventricular heterotopias and hypoxic lesions.	Not indicated	Normal

The MT treatment condition was carried out in a group therapy consisting with 4 children participating in each group. Assignment to the experimental group was therefore on the basis of order of arrival and availability of a slot for the MT group. All other children were assigned to the control group for neuro-restoration as usual (which was not a group therapy). This resulted in samples of 17 children (7 girls) for both the experimental and control group. We emphasize that a completely randomized subject allocation to the two treatment groups was not feasible, due to the choice of already mentioned convenience sampling. Nevertheless, the treatment allocation was concealed from the physician (AEP) in charge of initial interviews and obtaining parental consent and in accordance with RCT guidelines in MT (Bradt, 2012). In this study we employed only one music therapist at all times and who was blinded to the child's test results. The outcome assessors were speech and occupational therapists and the EEG technician who were responsible for measuring behavioral and physiological responses and all were blinded to the treatment group allocation of the patients. Furthermore, the assessors had no or minimal knowledge of the MT intervention, and carried out their evaluations in separate locations from one another and distant from the place where the patients underwent their therapeutic interventions.

During their first week at the hospital all patients received a multi-disciplinary evaluation and complementary examinations, such as EEG and structural imaging (1.5 T MRI), in order to establish a diagnosis and to propose individualized neurorestorative programs (Table 1). A comprehensive battery of standard psychometric and neuropsychological tests was employed to assess the neuropsychological impairments of patients according to their age and individual disabilities Progressive Matrices Test (Raven, 1938); Wechsler intelligence scale children WISC-r (Wechsler, 1974); Brunet-Lezine psychomotor scale (Josse, 1997); Children neuropsychological scale ENI (Rosselli-Cock et al., 2004). see Table 2 for the specific motor, cognitive, and language impairments from both groups.

**Table 2:** **Summary of deficits of the samples**.

**Impairment**	**Severity**	**Control group**	**Experimental group**
Psychomotor and mental retardation	Moderate to severe	9	10
	Mild	6	7
	Normal	2	0
Mobility	Dependent on wheelchair	10	5
	Walking aids	4	3
	Independent	3	9
Language	Poor or no language	8	5
	Normal	9	12
Attention	Moderate to severe	5	8
	Mild	9	7
	Normal	3	2
Praxis	Moderate to severe	10	12
	Mild	3	5
	Normal	4	0

The important point for clinical trials is to show changes of the outcome measures between the control and experimental groups. Here is most relevant in view of the wide range of cognitive and physical deficits in the children studied. The sequential recruitment of patient's (convenience sampling) precluded a priori calculation of the equivalence of the treatment groups and therefore of the outcome baseline scores before treatment, as is recommended in Bradt (2012). Nevertheless, a posteriori, the groups were found to be equivalent in clinical, social and demographic characteristics as described in the first section of the results. This heterogeneity also shaped the selection of statistical procedures that emphasized the measurement of intra-subject changes. The test battery applied allowed a *post-hoc* comparison between treatment groups to assess whether there were any baseline differences. All children were also subjected to an initial evaluation including a behavioral questionnaire and an Event Related Potential study (ERP).

### Neurorestorative program (NRP)

Here we describe the standard Neurorestorative Program (treatment as usual) which is applied to all subjects, irrespectively of belonging to the experimental or control group. Children were involved 7 h per day in different therapies (motor, language, occupational, physical stimulation and neuropsychology) and each therapy session lasted for a minimum of 1 h. The timetable of the therapies varied according to each patient's needs although the NRP was administered for a minimum of 4 weeks after which a first post-therapy behavioral questionnaire was applied. Depending upon the patient's needs and availability, the therapy then continued for another 4 weeks after which a second behavioral questionnaire was applied. In all cases a final ERP evaluation was carried out at the end of the therapy period (i.e., either 4 or 8 weeks).

While children in the control group did not receive any extra activity equivalent to MT, they were given more of the standard NRP instead.

### Music therapy (MT) protocol

We designed a specific MT protocol named Auditory Attention plus Communication which involved children listening to different musical excerpts and focusing their attention on specific aspects of the music (e.g., changing melody dynamics, rhythmic patterns). Two basic procedures in the Auditory Attention plus Communication therapy were designed to stimulate either sustained or selective attention. In the sustained attention procedure the child was required to throw a ball to another child in synchrony with changes in musical cues. In the selective attention procedure the child was required to focus on one instrument and ignore the others. The procedure manual for this protocol is provided in the Supplementary Material and is closely related to a standard Neurological Music Therapy Protocol described by Thaut and Hoemberg (2014) named “Musical Attention Control Training” (MACT).

Thus our protocol Auditory Attention plus Communication was designed to increase the levels of sustained and selective attention and verbal and nonverbal communication between children with diverse neurological disorders, using therapeutic games based on the properties of music and the benefits of group interactions. This is why the procedures were implemented as structured games and exercises for groups of children. Importantly, all actions were guided by the therapist and modulated by feedback on task performance. The responses requested from each child varied according to their level of disability. When the child had an upper limb impairment, he/she was requested to move another part of the body (head or shoulders) to signal a response to the music. They were then assisted to complete the task. A minority of children with severe mental retardation or impaired understanding were also helped to complete the task, but their effective engagement was evaluated using performance, rhythm and melody scales described in the procedure manual (Appendix 4 in Supplementary Material). To be considered as engaged, the child had to score more than 3 points on each of the scales. As described in results only three children scored 2.

Four different sequences of musical excerpts (each 1–2 min long) were prerecorded and each was used in different sessions to avoid habituation. The use of short excerpts rather than complete musical pieces was found to be more effective for maintaining attention in these type of children as determined by a pilot trial conducted in the same clinical settings (April to July 2012) with a sample of 17 pediatric patients (not included in this study). This pilot study also suggested that musical excerpts were best presented to a group of 4 children in a quiet, dedicated room, using a computer and external speakers. These musical pieces had different characteristics regarding rhythm, melody, intensity and timber. The complete list of excerpts can be found in the Procedure Manual.

In the present trial the Auditory Attention plus Communication protocol was applied in 10 min sessions immediately before the standard speech and occupational therapies, three times a day and on 3 days per week over 4 or 8 weeks depending on the duration of therapy. This resulted in a total of 36 sessions of MT (360 min) being given after 4 weeks and 72 sessions of MT (720 min) after 8 weeks.

One certified therapist (KMM) was in charge of administering the MT to all the children involved in the protocol.

### Behavioral outcomes

In order to explore a wide range of behavioral outcomes we designed a special purpose questionnaire that incorporated several different well established procedures. All items were scored on a 5 point Likert scale (1 = no reaction to 5 = relevant) and were completed by the occupational and speech therapists. The instrument was constructed by selecting items from standard and validated behavioral questionnaires that were most likely to reveal improvements in the motor, social, emotional, and cognitive domains: the MacArthur-Bates Communicative Development Inventories I and II, (Jackson-Maldonado et al., 2003); IDEA: inventario de espectro autista (Riviere, 2004); CUMANIN, (Portellano Perez et al., 2000). To keep the number of questions down to a manageable level, 5 experts selected by consensus a total of 23 items. A draft version of the questionnaire was explored during a previous pilot study. The reliability and validity of the final instrument (Appendix 1 in Supplementary Material) was assessed by means of multivariate item response theory (MIRT), using a “latent” variable (neuropsychological status) where each item was examined separately (see Data Analysis section).

Even though the therapists who completed the questionnaire knew about the existence of the MT program, they were blind to whether the children were in the experimental or control groups. There was no attempt made to guarantee that the same evaluator was used for any given patient. Importantly, this potential confound was incorporated in the statistical model described below where a random factor was included to control for evaluator variability.

### ERP mismatch paradigm

The ERP Mismatch Paradigm consisted in presenting a sequence of syllables (Consonant-Vowel structure) with the syllable “Ba” serving as a standard stimulus and deviant stimuli were vowel frequency, vowel duration and Voice Onset Time (VOT; the syllable “Pa”). The standard stimulus “Ba” had a fundamental frequency (F0) of 103 Hz, vowel duration of 208 ms and VOT of 70 ms, resulting in a total stimulus duration of 278 ms For frequency deviant syllables, the F0 of the vowel was increased to 155 Hz using Praat v 4.0 software (Boersma and Weenink, 2001). For duration deviant syllables, vowel duration was shortened by 75 ms using Adobe Audition resulting in a total syllable duration of 203 ms. Finally, for VOT deviant syllables the VOT was 70 ms shorter than for the standard syllable for a total duration of 208 ms.

Frequency, duration and VOT deviant syllables were semi-randomly intermixed with standard syllables (at least one standard syllable between the deviant ones) within the auditory sequence, with a fixed Stimulus Onset Asynchrony of 600 ms. A total of 920 stimuli were presented binaurally with 76% standard and 8% for each type of deviance. All stimuli were presented within a single block that lasted for 8 min.

The mismatch responses were obtained from EEG recorded continuously at a sampling rate of 200 Hz using a MEDICID IV amplifier system (Neuronic, Cuba) from 19 active Ag-Cl electrodes at standard positions of the International 10/20 System (Jasper, 1958): Fp1, Fp2, F7, F8, F3, F4, C3, C4, T5, T6, T3, T4, P3, P4, O1, O2, Fz, Cz, Pz, and nose. Data were filtered with a band-pass filter of 1–30 Hz (12 dB/oct) and transformed off-line to the Laplacian or current source density montage (Pascual-Marqui et al., 1988).

During EEG recordings, children were told to watch a silent movie without paying attention to the sounds that were presented through their headphones.

### Ethical safeguards

This study was carried out with the full support and supervision of the hospital and was approved by the hospital ethical committee. The project was conducted in accordance with the Declaration of Helsinki for the protection of the rights of human subjects. Parents of children included in the study were informed in detail of the procedure and music therapy program and signed an informed consent form.

## Data analysis

### Behavioral analysis

In accordance with recommended best practice for constructing outcome measures for neurological clinical trials, we employed Multivariate Item Response Theory (MIRT) to select informative items and to combine them into summarizing scores (Hobart et al., 2007). Potentially several outcome measures may be obtained from a given set of behavioral tests. Each outcome measure is a summarizing statistic obtained by selecting certain items from the complete set of behavioral tests, and adding them up each multiplied by a weight reflecting their importance for the outcome being probed. Thus the profile of items selected and weights chosen characterize each outcome as a latent variable designed to be independent of the evaluator, independent of the specific test items used in its construction, and robust against chance fluctuations in score recording (Fox, 2010). We must stress that MIRT finds optimal weights by means of a type of nonlinear principal component analysis. A more complete technical description of MIRT is contained in Appendix 2 in Supplementary Material.

Once the outcome measures are obtained, MIRT allows the application of mixed ANOVA (random + fixed) effects analysis of variance techniques to the underlying factors to query whether the proposed treatment actually affects the outcome measures.

In order to apply MIRT, the data from the behavioral questionnaire was arranged in a data matrix in which each observation (row) consisted of the scores for the 23 items. A separate row was used for each testing “Session” [Time0 = baseline, Time1 = 4, and Time2 = 8 weeks (if applicable)] and for each type of therapist (speech or occupational).

For the behavioral data, we addressed three statistical questions:

1. What is the adequate number of outcome measures that describe the variability of the behavioral data? One and two factor models were calculated using the *mirt* procedure (from the MIRT package) and the Bayesian Information Criteria (BIC) (Luo et al., 2013) measure was used to select between these models.
2. What items in the questionnaire contain useful information? This was decided on the basis of their factor scores (discriminatory power). Only high scoring items were retained for further analysis. Furthermore what is the interpretation of the outcome measures obtained by MIRT?
3. Was there a significant effect on outcome measures specific to MT? An ANOVA mixed-model including only the “Session” main effect and the interaction “Group by Session” analysis assessed this effect of therapy. The fixed effects considered were: Session (moment of evaluation) taken as a continuous regressor and Group: (experimental vs. control). The random effects considered were: Subjects (in a repeated-measures design) and Evaluators (different therapists).

### MMR analysis

The EEG was recorded from a subset of 24 children (12 out of 17–5 girls and 7 boys—in each group) in both the experimental and control groups (the first 12 rows in Tables 1A,B respectively). Children who required anesthesia for the initial EEG recordings (Time 0) were not included in the subsequent ERP analysis. Demographical and clinical characteristics did not differ significantly between the treatment groups for this subset of children. ERPs were recorded from all 24 children before (Pre-treatment) and after therapy (Post-treatment -either after 4 or 8 weeks), as indicated in Tables 1A,B, column 4. In order to take into account the duration of therapy for each child this variable was included as a covariate in all statistical analyses.

EEG recordings for each subject were segmented into trials from 100 ms before the stimuli (standard or deviant) to 500 ms. Artifact removal was carried out using the EEGLAB Matlab Toolbox (Delorme and Makeig, 2004) (http://sccn.ucsd.edu/eeglab/) as described in detail in Appendix 3 of Supplementary Material. The whole process resulted in sets of trials for each Session (pre/post therapy) and type of Stimulus (standard/deviant) with a range of 492–704 trials for the standard stimulus and a range of 48–72 for each type of deviant stimuli, with a total of 640–920 trials per subject.

The statistical analysis of the MMR response comprised the following steps:

1. Calculation for each individual of the Mismatch Response (MMR) computed as the average of deviant stimuli minus the standard stimuli for each session separately (Pre and Post).
2. Calculation for each individual of the Post- minus Pre-MMR i.e., the “MT difference MMR.”
3. Test for the “MT-specific effect” on the MMR by comparing the treatment difference MMR of the experimental and control groups. This was done by means of both a *t*-test as well as an ANCOVA analysis using duration of therapy (4 or 8 weeks) as a covariate. The latencies of MT specific effects were of particular interest.
4. Identification of the brain sources of MT-specific MMR effects. The LORETA procedure (Pascual-Marqui et al., 1994) was used to estimate EEG sources for each individual at significant MMR latencies. The significance of “MT-specific source effects” was independently verified by means of permutation techniques (Nichols and Holmes, 2001), and the anatomical locations of the sources were identified according to the AAL atlas (Automated Anatomical Labeling of Activations) (Tzourio-Mazoyer et al., 2002).

Steps 1–3 were carried out using a Mass Univariate approach (Groppe et al., 2011) as implemented in the LIMO (LInear MOdeling) Toolbox for Matlab (Pernet et al., 2011) http://www.gnu.org/software/octave/). LIMO carries out statistically robust (resistant to outlier) procedures by computing thresholds using an empirical distribution function based on 1000 bootstrapped samples and the use of Least Trimmed Squared (LTS) estimates. Finally, thresholds to deal with multiple comparisons were obtained by using the one dimensional temporal clustering correction. Step 4 was carried out with in-house software.

## Results

### Equivalence of treatment groups at baseline

Since the creation of the control and experimental groups was not guaranteed to be completely random, we did a *post-hoc* analysis to determine whether the major characteristics of both groups were homogenous. Mann-Whitney U tests were used to test for group differences. Results showed that the mean ages of the children in the two groups were not significantly different (6.83 ± 3.22 and 7.71 ± 3.94 years for the experimental and control groups respectively, *p* = 0.44). Family socio-economic background (assessed by income), as well as parental educational level (assessed by years of schooling), were also not significantly different (*p* = 0.91 and *p* = 0.96 respectively). There were also no differences in the neuropsychological impairments described in Table 2 between both groups. This was tested with a generalized linear model analysis (*p* > 0.87).

### MT therapy application

Compliance to the MT protocol was 100%. Of the 17 children that received MT treatment 14 scored more than 3 on each of the engagement scales. The other 3 children scored 2 but we decided to include them to avoid any bias in the statistical analysis.

### Behavioral results

Regarding the number of outcome measures, we found that only one outcome measure was necessary to describe the variability of the behavioral questionnaire. Since the questionnaire was constructed so that increasing scores reflected more wellbeing, the predominantly positive factor scores F1 (shown in Table 3) indicate that the outcome measure may be considered as the overall Neuropsychological State (NPS) of the children. Emphasizing, the statistical analysis provided by MIRT supported the usefulness of only one outcome measure.

**Table 3:** **Factor scores *F*_1_ for each item**.

	***F_1_***
Motor active	0.308
Attention	0.842
Sleepiness	0.144
Receptive	0.869
Reactive	0.745
Repetitive movements	0.240
Playing games	0.876
Interested and motivated	0.892
Calm and relaxed	0.561
Cooperative	0.909
Aware of other children	0.921
Spontaneous verbal communication with other children	0.955
Spontaneous non verbal communication with other children	0.854
Reactive verbal communication with other children	0.952
Reactive non verbal communication with other children	0.862
Spontaneous verbal communication with the therapist	0.970
Spontaneous non verbal communication with the therapist	0.801
Reactive verbal communication with the therapist	0.956
Reactive non verbal communication with the therapist	0.788
Happy	−0.377
Crying	0.528
Aggressive	0.626
Emotionally labile	0.483

*The items with F_1_ < 0.8 (highlighted) were not included in further analyses*.

**Figure 2:**
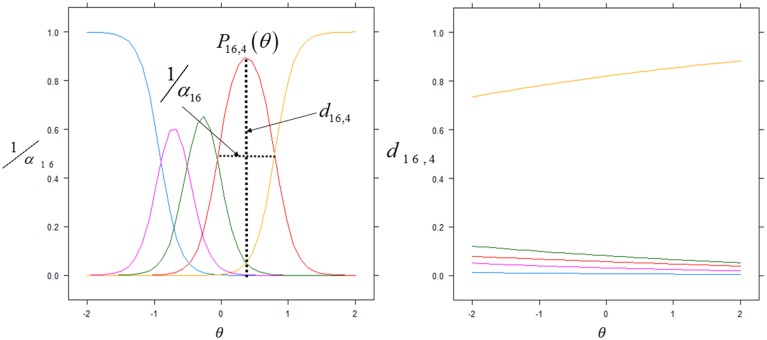
**Item discrimination profiles for two items**. The curves depict the probabilities of scoring a response at level *l* for item *j*: *P*_*j*, 1_(θ). The profiles on the left are those of item 16 (Spontaneous Non Verbal Communication with other Children) for which each level of the Likert scale responses exhibits good discrimination between scores. We highlight the probability density curve for the 4th level of this item and indicate both the discrimination and the intercept for this curve. The profiles on the right are those of item 3 (Sleepiness) which elicited highly unreliable scoring from the evaluators.

We next examined the usefulness of the items included in the initial behavioral questionnaire. Inspection of Table 3 shows two clusters, one with values of F1 < 0.80 and the other one with values of F1 > 0.80. Only the later were kept for further analysis (13 out of 23 items). The rationale for this selection was that by using the discrimination and offset parameters to determine the factor score it is possible to further evaluate the actual discriminatory power of each item. As can be seen from Figure 2 (left), items with the best discrimination power have higher estimated probabilities for extreme (1 and 5) than for intermediate values on the Likert scale. By contrast, items with low factor scores (Figure 2, right) do not show clear differences between the 5 levels of the Likert scale. It is important to point out that the selection criteria based on high factor scores is geared toward picking items that show clear separation of probabilities between the different levels of the Likert scale, and *not* between the two groups (control vs. experimental), since responses for all children were included in this analysis. Table 4 shows the discrimination and intercepts for the 13 questionnaire items selected.

**Table 4:** **Discrimination α_*j*_ and intercepts for each Likert score**.

**Item *j***	**α_*j*_**	***d*_*j*, 2_**	***d*_*j*, 3_**	***d*_*j*, 4_**	***d*_*j*, 5_**
Attention	2.621	6.073	3.964	1.902	−1.399
Receptive	2.934	4.739	2.035	0.1	−3.098
Playing games	3.066	4.123	2.691	0.641	−2.554
Interested and motivated	3.31	5.02	2.638	0.339	−2.508
Cooperative	3.728	4.47	2.884	0.658	−2.98
Aware of other children	4.067	7.603	5.229	2.714	−1.513
Spontaneous verbal communication with other children	5.84	4.887	2.889	−0.013	−4.075
Spontaneous non verbal communication with other children	2.751	2.279	0.74	−0.981	−3.643
Reactive verbal communication with other children	5.489	4.243	2.666	−0.438	−4.714
Reactive non verbal communication with other children	2.831	2.493	1.072	−0.83	−4.206
Spontaneous verbal communication with the therapist	7.161	6.699	3.774	0.558	−5.496
Spontaneous non verbal communication with the therapist	2.2	2.367	1.239	−0.539	−2.81
Reactive verbal communication with the therapist	5.443	5.985	3.334	−0.172	−4.078

It should be noted that the most frequent items were those measuring communication and interaction with other children.

**Figure 3:**
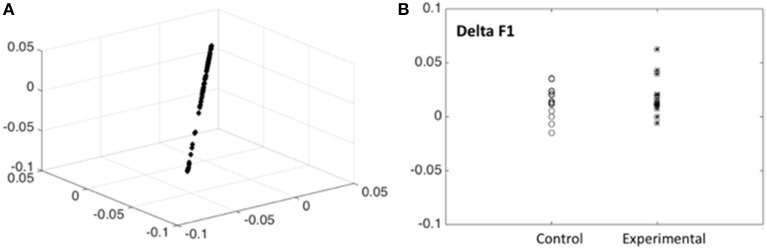
**Intrinsic dimensionality of the behavioral data. (A)** Scores for the 13 scores with good discrimination for all subjects are shown, plotted on the first 3 dimensions of the Laplacian Eigenmaps representation (Belkin and Niyogi, 2002). Note that the scores are all concentrated around a straight line indicating that a single outcome score (F1) for children is adequate. **(B)** Differences between post—pre scores (Delta F1) for the outcome measure (Neuropychological status) F1 shown for children from the control and experimental group. Note that the experimental group has, on the average, higher scores. Tables 3, 4, 5 substantiate the statistical significance of this graph.

In order to have an independent statistical validation of the existence of only one factor we explored the intrinsic dimensionality of the behavioral data by mapping all items onto a low dimensional space for visual inspection. The technique used for this purpose was that of Laplacian Eigenmaps representation (Belkin and Niyogi, 2002) which essentially compresses the 13 dimensional data points (one dimension item) for each subject and time of examination into only three dimensions but preserving the distances between individuals. The resulting plot (Figure 3A) shows that all data points are all essentially concentrated around a straight line. This constitutes an independent statistical validation of the fact that a single outcome score (F1) for children is adequate.

Possession of the factor scores allows examining the differences between post—pre conditions for this outcome measure, which we term Delta F1, which reflects improvement or decrease of the Neuropychological status. In Figure 3B the delta F1 are shown for children from the control and experimental groups. Note that the experimental group has, on the average, higher scores, indicating a definite effect of music therapy. We now substantiate the statistical significance of this graphical output by carrying out the appropriate mixed effects ANOVA to control for possible confounding factors.

Toward this end, the 13 remaining questionnaire items, were subjected to an ANOVA. There was no difference at baseline between the two groups in spite of the convenience sampling, supporting the validity of testing for effectiveness. Inspection of the ANOVA results (Table 5) shows that both a main effect of Session and a Group × Session interaction are highly significant.

**Table 5:** **ANOVA table for fixed effects of Session and Session:Group interaction**.

	**Coefficient**	**Standard error**	***Z*-value**	**One sided *P*-value**
**MIRT**				
**Session**	0.375	0.014	6.615	1.8578e-11
**Session: Group**	0.373	0.046	4.09	4.6476e-07

The main effect of Session confirms a general beneficial effect of the Neurorestoration program applied to both groups. Importantly, the interaction between Group and Session indicates a differential effect of Musical Therapy compared with standard Neurorestoration therapy alone. In fact this differential effect is quite strong since the slope of the MT group (*z* = 12.614, *p* < 0.001) is nearly twice that of the control group (*z* = 6.84, *p* < 0.001).

### ERP mismatch response results

**Figure 4:**
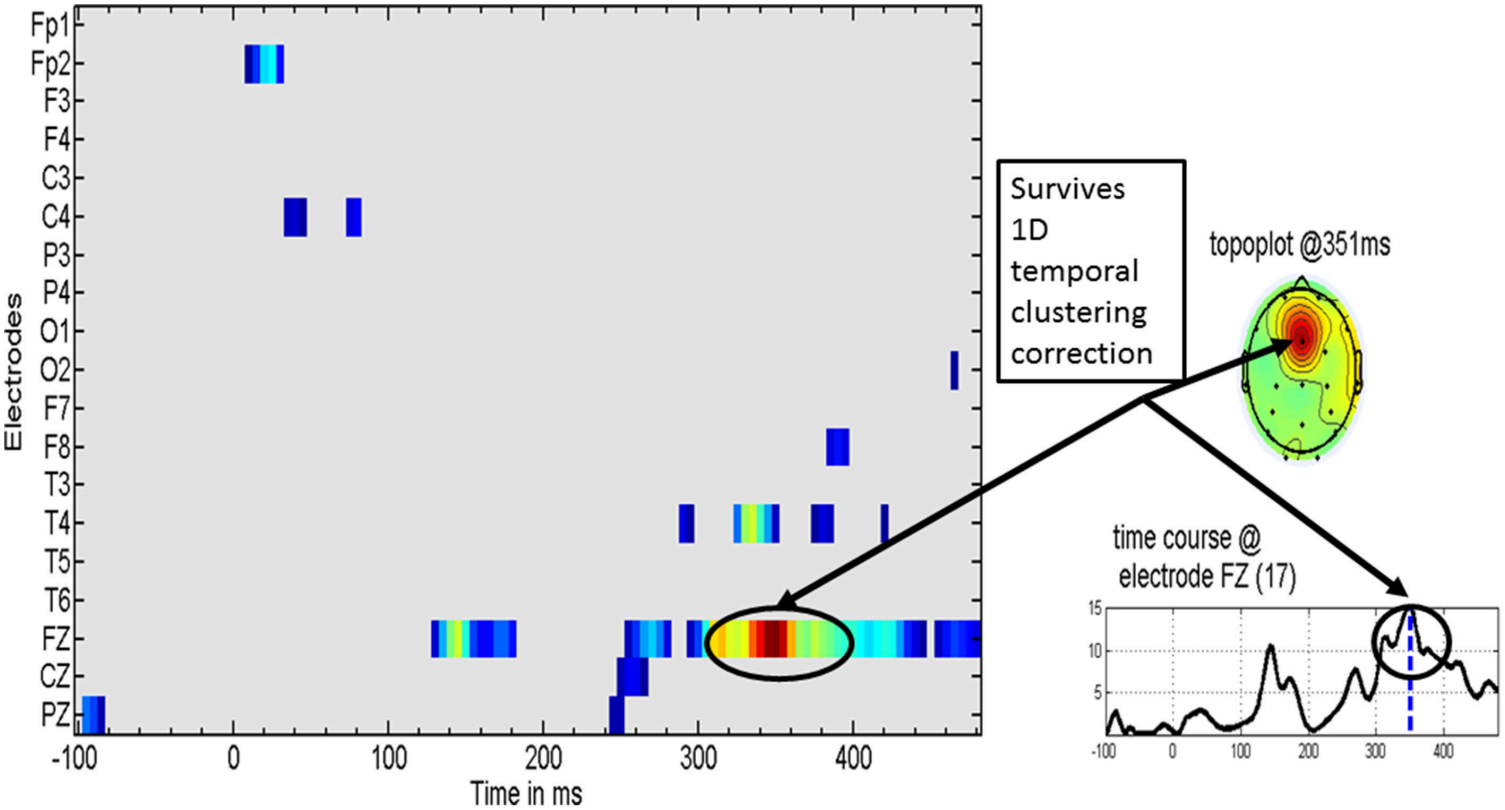
**Specific music therapy treatment effect on the mismatch response**. (Left) Plot of MT treatment effect for each EEG derivation (y axis) and time (x axis). The *t*-values are thresholded at the uncorrected univariate for *p* = 0.01 level. (Right top) Topography of the *t* statistic at the most significant time point. (Right bottom) the t waveform for the most significant derivation (Fz). Highlighted with circles is the time interval for which the *t*-tests correction for multiple comparisons was significant. The dashed line for the t waveform indicates the maximum significance at 351 ms.

Figure 4 shows the distribution of significant *t*-test values for the specific effect of MT on the MMR. These evidence a clear effect at the Fz derivation in the latency range of 308–400 ms, with a peak at 351 ms.

**Figure 5:**
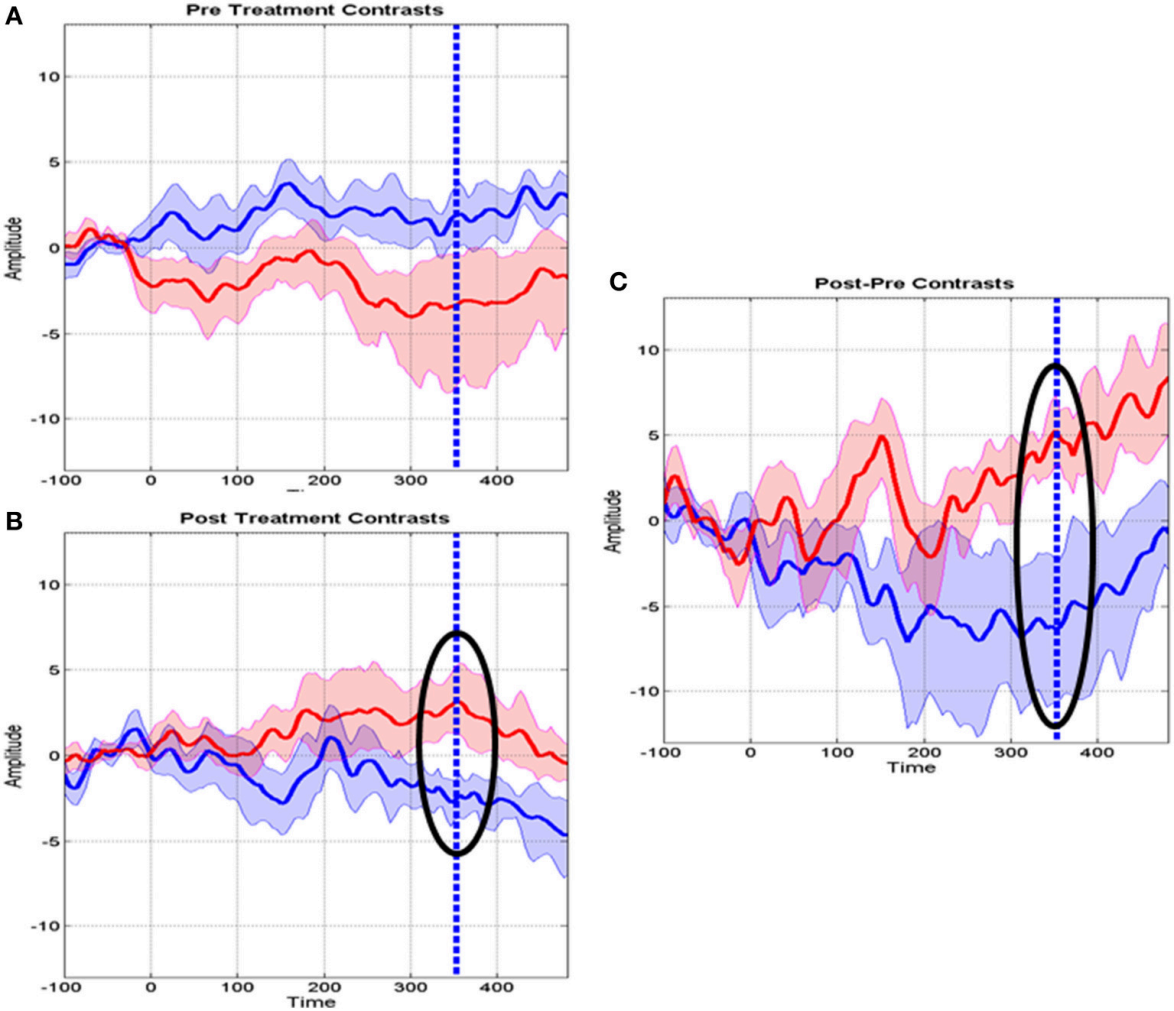
**Means and standard deviations of mismatch responses (MMR) at the Fz derivation. (A)** Baseline MMR for the Control (blue) and MT groups (red). **(B)** Post-treatment MMR for the control and MT groups. Highlighted with a circle is the interval where the t tests survived correction for multiple comparisons. The dashed line indicates maximum significance. **(C)** Post-Baseline treatment MMR. Circles and dashed lines as in **(B)**.

Further details about MT specific changes at Fz are provided in Figure 5 which shows the means of several contrasts and their 95% confidence intervals. The baseline MMR for the experimental and control groups did not differ significantly (Figure 5A) whereas the post-MMR (Figure 5B) shows the same type of changes as in the treatment specific comparison of Figure 4. We ruled out possible bias with therapy duration (4 vs. 8 week) with an ANCOVA (not shown) including the duration of therapy as a covariate. Figure 5C shows the post-pre contrast, once again confirming the time range of 308–400 as containing the discriminative ERP changes.

**Figure 6:**
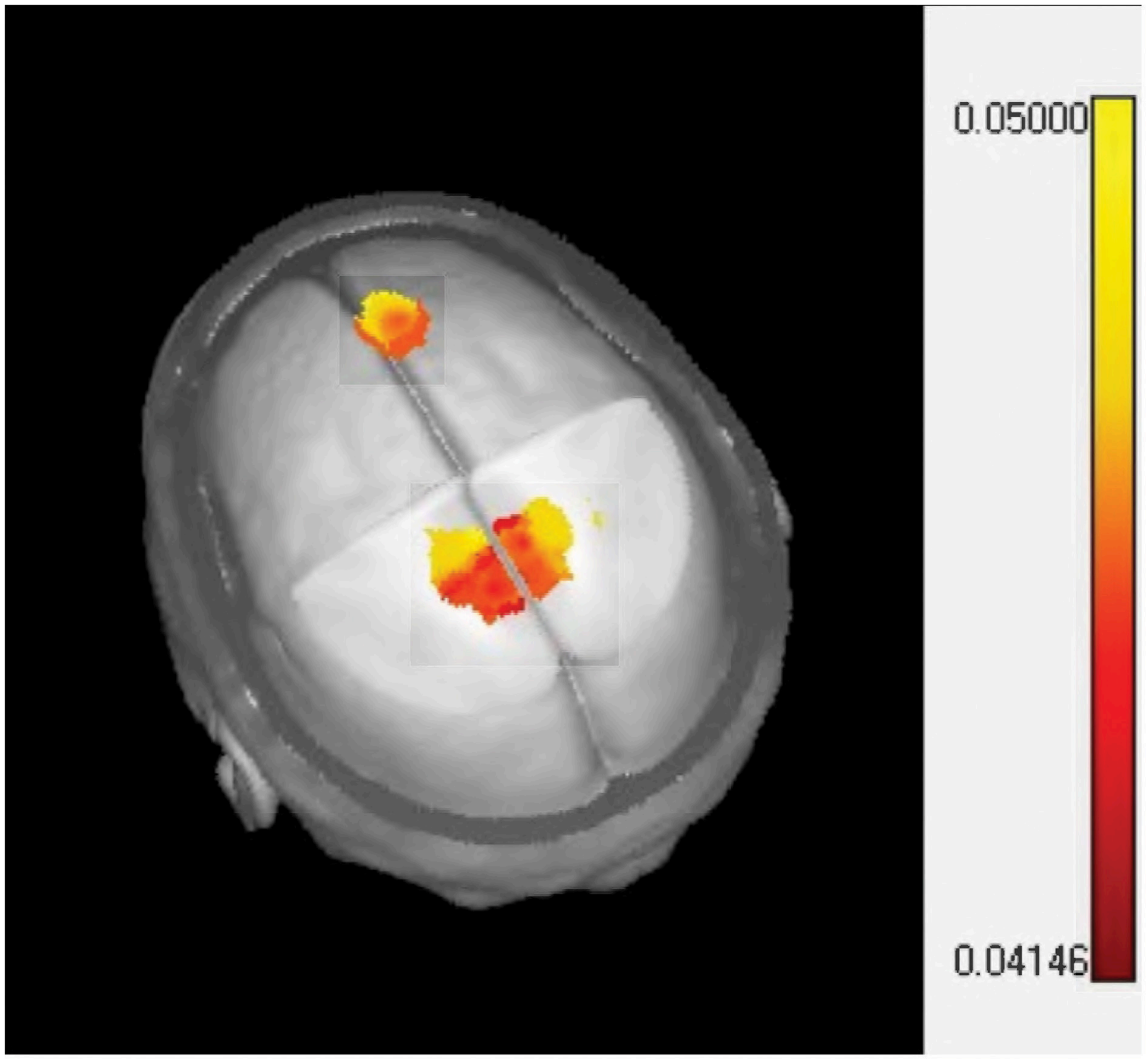
**Sources localization of the music therapy specific mismatch responses**. The one sided *t*-test of LORETA images plotted on the MNI brain template identifying significant activation in the midcingulate (bilateral) and prefrontal cortex.

Source analysis of the MMR identified two specific regions (at the latency of 351 ms) that significantly increase activity when MT is given in addition to standard neurorestoration therapy (*p* = 0.041). These regions were the right prefrontal cortex and the bilateral medial cingulate cortex (Figure 6).

## Discussion

Our results support an affirmative response to both of our main research questions. Firstly, we provide evidence for the effectiveness of MT in addition to standard neurorestoration therapy in real world situations. Secondly, we also demonstrate MT-specific changes in brain plasticity as reflected by enhanced Mismatch Responses. We now discuss these two results in more detail.

### Effectiveness of MT in neurorestoration settings

The results with the MIRT analysis show highly significant effects that are specific to the addition of the “Auditory Attention plus Communication” MT protocol to the standard neurorestoration therapy. It can be argued that the between-group differences reported here reflect the influence of having an additional therapy independent of its content (i.e., the effects are not specific to the MT program). While the constraints imposed by the clinical settings did not allow us to include a control group with another therapy that would be as motivating for the children as MT, the present results show that adding MT to the standard neurorestoration program is more beneficial to the children than having more of the standard restoration program alone. This supports our conjecture that rather than a “greater dose” of a specific neurorestoration therapy as advocated by Forsyth and Basu (2015), a different type of therapy can also have an enhancing effect.

When reviewing prior studies we noted that these refer to *efficacy* of MT in *specific* pathologies. An example is the efficacy of music therapy with autistic children as reviewed by Geretsegger et al. (2014). In contrast we have demonstrated here MT's *effectiveness* in a real world setting across a heterogeneous patient sample. This is probably due to the use of the MIRT analysis, a more powerful statistical procedure than conventional approaches based on the analyses of summarized scores and without detailed analysis of the contribution of each questionnaire item.

The purpose of this protocol is to facilitate training in other nonmusical domains, such as attention and communication. It is therefore interesting to note that most of the items that describe the improvement of neuropsychological status are precisely those that measure these domains. This is consistent with work that shows music training enhances perceptual (auditory) and cognitive (attention, short-term memory and executive) functions as well as sensori-motor associations (see Janata et al., 2002; Kraus and Chandrasekaran, 2010; Besson et al., 2011; Schellenberg, 2011; Rodriguez-Fornells et al., 2012). Music training also stimulates brain plasticity in several brain regions (Münte et al., 2002). Moreover, the MT program clearly enhances children's motivation and social behavior, thereby accounting for the specific improvements in communication, cooperative behavior and awareness of other children (see Koelsch, 2014 for a review of the effects of music—evoked emotions on the brain). In summary, we surmise that MT promotes brain changes in areas related to both attention and emotional responses with a consequent influence on communication and social interactions. We will now discuss support for this hypothesis from our current electrophysiological findings.

### Brain plasticity changes in MT reflected by ERP mismatch responses (MMR)

We found that the ERP MMR response is sensitive to MT, which is not surprising in view of previous ERP studies related to music training. The best known of the MMR family is the classical “early negativity” first identified by Näätänen et al. (1978). Related to this classical response, Chobert et al. (2014) showed that musical training increases the classical early negative MMN reflecting pre-attentive training.

In contrast the responses we found rather than being of the classical type are of the “late” MMR type described by Korpilahti et al. (2001) and the “positive” MMR type as described by Dehaene-Lambertz and Dehaene (1994). Related to this type of response Putkinen (2014) showed in a longitudinal study that musical training in healthy children enhances later attention-related functions with corresponding positive MMR changes. All the studies cited are with healthy children and only referred to topographic localization, which is brain activity reflected on the scalp. This kind of measure is difficult to relate to brain regions engaged in the response to MT.

For this reason, we further analyzed the MMR responses in order to identify the brain areas that might generate the observed MT-specific effects. The two areas identified using LORETA are consistent with the neural systems we designed to be influenced by the Auditory Attention plus Communication therapy:

1. The medial cingulate cortex is a caudal “cognitive” division of the anterior cingulate cortex defined by Bush et al. (2000) which sends projections to the lateral prefrontal cortex. It is a component of a distributed attentional network activated by cognitively demanding tasks (e.g., Stroop, flanker tasks) and which is also involved in performance monitoring, mismatch detection and feedback processing (Bush, 2009; Shackman et al., 2011). This area is also involved in processing and perception of pain, emotion, stimulus salience, action-reward associations, and premotor functions among others (Rushworth et al., 2007).
2. The prefrontal areas are particularly associated with cognitive functions (attention, working memory) and the dorsolateral prefrontal cortex is activated during tasks requiring executive function (Kane and Engle, 2003), e.g., regulation of encoding, strategy selection, and manipulation and retrieval of information. Park et al. (2014) have also reported that neuro-affective processing of sadness and fear are modulated by musical training in right frontal regions.

To our knowledge this is the first study that shows brain plasticity induced by MT in neurologically compromised children using electrophysiological source reconstruction analysis.

### Limitations

While the results presented are encouraging there are a number of points which must be improved to sharpen the interpretation of these results. In the first place a larger study with effective randomization is required which can be designed on the basis of this study. The statistical analysis was carried out separately for behavioral outcomes with MIRT, and MMRs with the general linear model. These two types of responses should be analyzed in a common framework. For this purpose work is in progress to match individual MMR and behavioral outcomes. Also, even when the source localization of the MMR suggested functionally meaningful areas, they must be verified by other techniques with higher spatial resolution such as fMRI. Finally, the analysis of brain activity should not be limited to the detection of activation but would benefit by the identification of the neural networks involved by means of connectivity analysis (Valdes-Sosa et al., 2011). The actual involvement of the brain areas proposed and the effect on emotion and communication must, of course, be subject to an intervention trial.

## Conclusions

We have confirmed the effectiveness of a protocol for music therapy in addition to standard neurorestoration therapy as reflected in non-music performance outcome measures. The therapeutic effects of MT were demonstrated by improved attention and communication across a range of neurologically impaired children. Moreover, the ERP mismatch paradigm used evidenced differential changes in brain plasticity that were specific to MT and occurred at later latencies. This study can help to diminish the large “gap of evidence regarding the neurophysiological changes associated with applying the music as therapy” called for by Stegemöller (2014). Larger and completely randomized studies are warranted.

## Author contributions

MLB and MB designed the study. MO, RG, CM, and HV evaluated the children. LM and MZ carried out the electrophysiological recordings. DC was in charge of collecting the behavioral data. AP was in charge of interactions with the parents. KMM was in charge of the music therapy program. Statistical analysis was designed by PVS and carried out by MLB, PVS, LG, and PR. Finally, MLB, MB, and PVS wrote the paper.

### Conflict of interest statement

The authors declare that the research was conducted in the absence of any commercial or financial relationships that could be construed as a potential conflict of interest.
