# Supplementary material for: Effectiveness of music therapy as an aid to neurorestoration of children with severe neurological disorders
Source: Front Neurosci. 2015 Nov 4;9:427. doi: 10.3389/fnins.2015.00427 (PMC4631829; doi:10.3389/fnins.2015.00427)
Supplement: Supplementary file 1 [file DataSheet1.DOCX]

# Appendix 1: Behavioral Questionnaire for Speech and Occupational Therapists

**Name of patient: ______________________ Name of therapist: ________**

**Evaluation: At the beginning __ 4 weeks __8 weeks __ Date: __________**

**Please fill below your global appreciation of the patient during the therapy.**

| **General behavior of the child** | | **1. No** | **2. A few times** | **3. Sometimes** | **4. Very often** | **5. A lot** |
| --- | --- | --- | --- | --- | --- | --- |
|  | Is s(he) showing active movement? |  |  |  |  |  |
|  | Is s(he) alert to environmental stimuli? |  |  |  |  |  |
|  | Is s(he) sleepy or somnolent during the therapy? **^** |  |  |  |  |  |
|  | Is s(he) receptive to instructions about how to perform the tasks? |  |  |  |  |  |
|  | Does s(he) react and orient attention to sounds (when a door is open, or a phone rings)? |  |  |  |  |  |
|  | Does s(he) make persistent and repetitive movements (tics, stereotypy, balancing)? **^** |  |  |  |  |  |
|  | Does s(he) happily accept participation in interactive games? |  |  |  |  |  |
|  | Is s(he) motivated by and interested in the activities? |  |  |  |  |  |
|  | Is s(he) calm and relaxed? |  |  |  |  |  |
| ***Social Behavior*** | | **1. No** | **2. A few times** | **3. Sometimes** | **4. Very often** | **5. A lot** |
|  | Does s(he) show cooperation in group activities? |  |  |  |  |  |
|  | Is s(he) aware of other children? |  |  |  |  |  |
|  | Does s(he) exhibit spontaneous VERBAL communication with other children? |  |  |  |  |  |
|  | Does s(he) exhibit spontaneous NON VERBAL communication with other children? |  |  |  |  |  |
|  | Does s(he) exhibit reactive VERBAL communication with other children? |  |  |  |  |  |
|  | Does s(he) exhibit reactive NON VERBAL communication with other children? |  |  |  |  |  |
|  | Does s(he) exhibit spontaneous VERBAL communication with the therapist? |  |  |  |  |  |
|  | Does s(he) exhibit spontaneous NON VERBAL communication with the therapist? |  |  |  |  |  |
|  | Does s(he) exhibit reactive VERBAL communication with the therapist? |  |  |  |  |  |
|  | Does s(he) exhibit reactive NON VERBAL communication with the therapist? |  |  |  |  |  |
|  | ***Emotional Behaviour*** | **1. No** | **2. A few times** | **3. Sometimes** | **4. Very often** | **5. A lot** |
|  | Is s(he) happy during the therapy? **^** |  |  |  |  |  |
|  | Is s(he) crying during the therapy? **^** |  |  |  |  |  |
|  | Is s(he) irritable or aggressive during the therapy? **^** |  |  |  |  |  |
|  | Is s(he) emotionally labile, exhibiting rapid mood changes?**^** |  |  |  |  |  |
| Note: the items marked with **^**  have the inverse direction of the score. | | | | | | |

# Appendix 2: Multivariate Item Response Theory

This section explains the method used for the analysis of the behavioral outcomes: Multivariate Item Response Theory (MIRT). For each subject $i$ we assume an underlying set of latent variables $\theta_{i,m} m=1,\cdots,M$ that measure Neuropsychological State (NPS). These values can be arranged into the vector: $\mathbf{\theta}_{i}=\left(\theta_{\mathrm{i},\mathrm{m}}\right)_{1\leq l\leq M}$[[1]](#footnote-1). MIRT models the responses $x_{ij}$of the evaluators to each item $j$for subject $i$as a linear function of the NPS that also takes into account evaluator uncertainty. Assuming a binary response for item$j$, yes (1) or no (0), the cumulative probability density of a 1 would be a logistic type function of , where $d_{j}$is the point of the curve where the cumulative probability of response is 0.5 and the are the slopes of the logistic functions for each latent variable. The parameter $d_{j}$ is known as the intercept (maximum of the probability density) of the item and the slopes measures the discrimination power of the item.

In our specific case, using a Likert scale for each item, we apply Samejima's (Samejima, 1969) multidimensional ordinal response model. For each item $j$ there are $l=1\cdots5$ unique responses, each of which now have associated an intercept $d_{j,l}$, which may be arranged in vector $\mathbf{d}_{j}=\left(d_{j,l}\right)_{1\leq l\leq5}$. The probability density of $x_{ij}$ given NPS $\mathbf{\theta}_{i}$ will now be:

$$\begin{matrix} \Phi\left(x_{ij}\geq0\left|\mathbf{\theta}_{i},\mathbf{\alpha}_{j},\mathbf{d}_{j}\right.\right)=1 \\ \Phi\left(x_{ij}\geq1\left|\mathbf{\theta}_{i},\mathbf{\alpha}_{j},\mathbf{d}_{j}\right.\right)=\frac{1}{1+\exp\left[\mathbf{\alpha}_{j}^{T} \mathbf{\theta}_{i}+d_{j,1}\right]} \\ \vdots\\ \Phi\left(x_{ij}\geq l\left|\mathbf{\theta}_{i},\mathbf{\alpha}_{j},\mathbf{d}_{j}\right.\right)=\frac{1}{1+\exp\left[\mathbf{\alpha}_{j}^{T} \mathbf{\theta}_{i}+d_{\mathrm{j},l}\right]} \\ \vdots\\ \Phi\left(x_{ij}\geq5\left|\mathbf{\theta}_{i},\mathbf{\alpha}_{j},\mathbf{d}_{j}\right.\right)=0 \end{matrix}$$

Therefore the probability of obtaining a response equal to $l$ is

$\begin{matrix} P\left(x_{ij}=l\left|\mathbf{\theta}_{i},\mathbf{\alpha}_{j},\mathbf{d}_{j}\right.\right)=\Phi\left(x_{ij}\geq l\left|\mathbf{\theta}_{i},\mathbf{\alpha}_{j},\mathbf{d}_{j}\right.\right)-\Phi\left(x_{ij}\geq l+1\left|\mathbf{\theta}_{i},\mathbf{\alpha}_{j},\mathbf{d}_{j}\right.\right) \\ =P_{j,l}\left(\mathbf{\theta}_{i}\right) \end{matrix}$.

Examples of these curves for actual items can be seen in Figure 2. Note that the intercepts $d_{j,l}$are the values of the NPS for which the probability density of scoring $l$ on the Likert scale is maximum for item $j$.

It is to be noted that estimating the set of slopes (discriminations) $\mathbf{\alpha}_{j}$ may be interpreted as a type of nonlinear factor analysis and can, in fact, be transformed to be interpretable as traditional factor scores $\mathbf{F}=\left(F_{1},\ldots,F_{M}\right)$ using a procedure described in (Bock, et al. 1988). The number of latent variables $M$ is therefore analogous to the number of factors in Factor Analysis.

The Maximum Likelihood estimators for these models and for all procedures subsequently described were obtained using the R Package ***MIRT*** (Chalmers, 2014) available at the CRAN repository (<http://cran.r-project.org/> ) and further documented at <https://github.com/philchalmers/mirt>. , specifically the *mirt* procedure.

It is also possible to include in the model, and test for, fixed and random effects by using the *mixedmirt* procedure of the MIRT package. This augments the model described above by modeling the probability density of response $x_{ij}$as:

$$\Phi\left(x_{ij}\geq1\left|\mathbf{\theta}_{i},\mathbf{\alpha}_{j},\mathbf{d}_{j}\right.\right)=\frac{1}{1+\exp\left[\mathbf{\alpha}_{j}^{T} \mathbf{\theta}_{i}+d_{1}+\mathbf{y}_{i}^{T} \mathbf{\beta}+\mathbf{z}_{i}^{T} \mathbf{\gamma}\right]}$$

Where $\mathbf{y}_{i}$is the set of fixed design covariates and $\mathbf{z}_{i}$ the set of random design covariates respectively associated with observation$i$. The use of this mixed effects multivariate item response model will be described in the results.

**References**

Bock, R. D., Gibbons, R., and Muraki, E. (1988). Full-Information Item Factor Analysis. *App. Psych. Meas.* 12(3), 261–280.

Chalmers, P. (2014). *Package “ mirt”*. URL <http://cran.r-project.org/web/packages/mirt/mirt.pdf>

Samejima, F. (1969). Estimation of Latent Ability Using a Response Pattern of Graded Scores. *Psychometrika Monographs,* 34(4).

# Appendix 3: Stages of EEG artifacts rejection using EEGLAB

1. For each trial bad channels detected by visual inspection were substituted by interpolation using an in house plugin.
2. Identification and elimination of artifactual Independent Components (IC) was carried out with the EEG plugin ADJUST (Mognon et al., 2011) based on combining stereotyped artifact-specific spatial and temporal features. Features were optimized to capture blinks, eye movements and generic discontinuities. Once artifactual IC were identified, they were removed from the data by means of careful consideration of scalp distribution as well as time and frequency domain properties and the examination of the effect of purported artifact removal on the EEG traces, ERP and Spectra of the subject in all conditions. Using this technique is was possible, for example, to discard most of sleep spindle activities.
3. As a final stage the trials were analyzed using the EEGLAB plugin MARA ("Multiple Artifact Rejection Algorithm") (Winkler et al., 2014) which automates the process of hand-labeling independent components for artifact rejection.
4. Artifact removal was carried out by consensus of 3 specialists in signal processing under the supervision of MLB who carried this out blindly without consideration of type of stimuli or subject characteristics.

**References**

Mognon, A., Jovicich, J., Bruzzone, L., and Buiatti, M. (2011). ADJUST: An Automatic EEG artifact Detector based on the Joint Use of Spatial and Temporal features. *Psychophysiology* 48(2), 229–240.

Winkler, I., Brandl, S., Horn, F., Waldburger, E., Allefeld, C., and Tangerman, M. (2014). Robust artifactual independent component classification for BCI practitioners. *J. Neural Eng.* 11(035013).

# Appendix 4: Manual for applying Music Therapy with the protocol: Auditory Attention plus Communication “AAC”

Authors: Maria L. Bringas^1,2^, Karelia Martinez-Montes^1^, Mireille Besson^3,4^

^1^International Center for Neurological Restoration CIREN La Habana Cuba, ^2^University of Electronic Sciences and Technology of China, UESTC, Chengdu China, ^3^Cuban Neuroscience Center CNEURO La Habana, Cuba, ^4^Centre National Recherche Scientifique CNRS Marseille, France.

## Definition:

The Auditory Attention plus Communication protocol (**ACC**) involves children listening to the different musical excerpts and focusing their attention toward specific aspects of the music (e.g. changing melody dynamics, rhythmic patterns). The two basic procedures of the AAC therapy are designed respectively to stimulate sustained and selective attention. Engagement is measured objectively with behavioral scales. The procedures and scales are detailed below.

**ACC** has been conceived to increase the levels of attention and communication between children with diverse neurological disorders using therapeutic games based on the properties of music and the benefits of group interactions. It is designed to be applied in established neurorestoration settings and just before other cognitive therapies in order to enhance performance of the latter.

Attention is the ability to select and focus on a given mental or behavioral task. It is a prerequisite for other cognitive processes (Posner 1988) and one of the most seriously impaired in neurological disorders. In neurorestorative settings it is evident, either by simple observation or neuropsychological assessment, that children with neurological disorders exhibit difficulties with attention. It is assumed that music, due to its nature and structure (complex sensory stimulation), has the possibility to engage children and, when applied systematically using structured active or receptive musical exercises, may improve attentional skills. Thaut (2005) pointed out that the musical cue can be a platform to develop attentional process “when linked with non-musical information,… adds structure and organization, emotion, and appeal to the information in order to increase the probability that attention will be focused, maintained, and/or switched”. Attentional processes can be: automatic versus controlled, effortful, and conscious. The automatic process involve bottom-up processing and the conscious processes: top-down processing. Our objective is increase the controlled process using exercises addressed to the sustained and selective functional components of attention.

Communication is also affected in these children either due to multiple impairments.

The two basic procedures of the **AAC** therapy target both sustained and selective attention as well as to increase verbal and nonverbal communication between patients. This is why the procedures are implemented as structured games and group exercises to enhance interactions between children. These actions are guided by the therapist and modulated by feedback about task execution.

## Target population:

Children with neurological disorders, aged between 3 and 12.

## AAC therapeutic exercises

### Procedure 1: sustained attention

**Sustained attention** (also called alerting) consists of vigilance, arousal, and the ability to sustain attention to a specific stimulus.

The therapist asks one children to give an active and overt response to the music change, throwing a ball to the closest child, who receives it.

The overt response to the change varies according the individual disabilities:

1. Children cognitively intact with functional upper limbs are able to identify the task demands and perform the action.
2. Children with intact understanding but with upper limbs impairment (because dystonia, quadriparesis and spasticity), are requested to move other part of the body (head or shoulders) to signal the change of the music. They will be assisted to complete the task.
3. Children with severe mental retardation or impaired understanding are also helped to complete the task but their effective engagement is evaluated using additional measures explained in the last paragraphs.

During the session all the children in the group must be engaged with the activity. Even the children waiting for their turn to grab or throw the ball, are instructed to clap hands or moving other body parts in conjunction with the musical cue.

When the child cannot use her hands due of motor disabilities, the therapist must place the ball in their chest and throw the ball together with her in order to guarantee motor performance and completion of task.

The objective of this procedure is for the patient to successfully pass a ball to a peer at least 5 out of 8 times during a 10 minutes session

### Procedure 2: selective attention

Selective attention (also called executive control/focused) involves the engagement of more complex operations, selection of relevant stimuli, and resolution of competing goals.

Children while listening to musical excerpts are asked to identify only one instrument and ignore others. When the instrument target appeared they are asked to signal the moment, clapping or using hands to simulate how to play it, if applicable. Another exercise involves dividing the group into two pairs. Each pair must identify a specific instrument while listening to musical excerpts involving several. An important element is the spontaneous error-correction feedback between members of a pair, the more cognitively preserved helping those with disadvantage. Finally the therapist mimics playing a musical instrument with his hands--with and without music-- and the children with preserved language call out the name of the instrument.

The minimum number of target instruments to identify by each child during a session is at least 50%. The children who can’t achieve this goal, are encouraged to do so during the remaining session of the day.

## Setting:

A small group (e.g. 4 children) is trained to work simultaneously during the daily sessions. It’s recommended to be an even number to facilitate working in pairs.

## Location and equipment:

The MT is delivered in a quiet, dedicated room with sound attenuation, using a computer and external speakers, on which the musical excerpts are prerecorded. Depending on the individual’s level of mobility, children can either stand or sit on a chair. If they are more impaired they may stay in their wheelchair.

## Musical stimuli:

At least four different sequences of musical excerpts are prerecorded and each one was used in different sessions to avoid habituation.

MT sessions are suggested to be brief, for example 10 minutes since they will be followed by neurorestorative therapy as usual. The session must have a predictable structure:

- Start with soothing music excerpts in order to introduce the session with the therapist
- Continue with more dynamic and engaging musical excerpts which are used to evoke overt responses during the tasks used to stimulate sustained and selective attention process
- End with more soothing excerpts and a brief relaxation exercise (closing eyes and slow movements) used to calm the children for the subsequent therapies.

The musical pieces we have used had different characteristics (rhythm, melody, intensity and timber) and were selected based on a pilot study conducted in the same clinical settings (April to July 2012) with a sample of 17 pediatrics patients (the list of musical pieces is available upon request and included excerpts from classical and popular music). The list of excerpts actually used is posted at the end of the manual but is only a suggestion.

## Duration and frequency of MT:

Typical ACC MT interventions involve 10 minutes sessions before cognitive therapies. In our experience this is possible up to 2 or 3 times a day, three days per week during 4 or 8 weeks according to the duration of therapy

## Behavioral scales for evaluation of engagement during therapy:

The level of performance of the children and their engagement during the MT was evaluated by the music therapist using different tools:

***Performance Scale:*** this scale comprises 5 domains: motor ability, attention, emotion, imitation and communication. At the end of each MT day, the therapist must fill a 5 points scale for each child (from 0 -poor level of performance- to 5 -high level of performance). Averaged individual scores are to be computed weekly (i.e., over 9 MT sessions). In addition, anecdotal notes are to taken of the individual behavior of the children in order to complete the evaluation. Examples of observations to be noted are the child’s facial expressions and gestures indicating liking of the music. Also their actions such as dancing, singing, clapping, beating the floor or seat to follow the rhythm of the music.

***Rhythm test*** (subtest of CUMANIN, Portellano Perez, Mateos Mateos, Martinez Arias, Tapia, & Granados, 2000):children have to reproduce a rhythmic sequence played by the therapist by clapping her hands. The number of taps and their grouping increase in difficulty (from level 1 to 7). Rhythmic performance was evaluated using a 5 points scale (0 poor and 5 exact reproduction). For data processing, a score equal or superior to 3 was considered as an index of successful rhythmic reproduction.

***Melody test***: this test was developed by coauthors KMM & MB to evaluate the orientation response, perceptual discrimination and attention. Fifteen music excerpts are presented twice to the children, once in its original version and once in a modified version that included an abrupt change in timber. This test is presented twice, under implicit and explicit conditions. In the implicit condition, the music therapist watch the child reaction 1 (no reaction) and 5 (child clearly reacted to the change). In the explicit conditions, children are given the following instructions: “We are going to play a little game. You listen to the music and you raise a finger/arm/foot/ leg/move your head, if you hear something surprising”. Scores varied from 1 (no reaction) to 5 (detect surprising event at the right time).

The Rhythm and Melody tests are administered to each child individually at different time points during the therapy (initial, final, and intermediate points according to the duration of the MT). Total scores must achieve a threshold of 3 to consider the child engaged. The scales can be obtained by request to the authors.

## References:

Posner, M. I., Petersen, S. E., Fox, P. T., & Raichle, M. E. (1988). Localization of cognitive operations in the human brain. *Science*, *240*(4859), 1627-1631

## Thaut, M.H. (2005). Rhythm, music and the brain: scientific foundations and clinical applications. New York: Routledge*.*

Portellano Perez, J. A., Martinez Arias, M. del R., & Mateos Mateos, R. (2000). Cuestionario de Madurez Neuropsicológica Infantil, CUMANIN. TEA EDICIONES.

## List of some music excerpt employed at the MT

- Carmen. Habanera. (Bizet)
- The Toreadors. (Carl Orff)
- Carmina Burana. (Carl Orff)
- Bolero (Ravel)
- Khachaturian, Aram “Toccata” (Jeff Abrams)
- “Four Seasons” (Vivaldi).
- Arb YA-MSAHARNI.
- Arb ZIKRAYATE.
- “He venido a decirte” (Los Zafiros).
- “Canta lo Sentimental” (Los Zafiros).
- “Bossa Cubana” Sexto Sentido.
- “Love is blue” (Frank Pourcel).
- “Aranjuez Mon Amour” (Frank Pourcel).
- “Air” Bach.
- Myrthenblueten (Myrtle Blossoms, opus 395) Strauss.

1. Scalars will be denoted by lowercase letters, vectors by uppercase. $\mathbf{x}^{T}$ denotes the transpose of the column vector $\mathbf{x}$. [↑](#footnote-ref-1)
